# A Perturbation Subsampling Method for Massive Censored Data

**DOI:** 10.3390/e28040476

**Published:** 2026-04-20

**Authors:** Yan Tian, Jiaxin Song

**Affiliations:** School of Mathematics, Liaoning Normal University, Dalian 116081, China

**Keywords:** Cox proportional hazards model, large-scale survival data, perturbed subsampling method

## Abstract

With the advancement of information technology, large-scale data have become increasingly common. Subsampling methods for the statistical analysis of such data require computing the sampling probability for each observation, a process that can be computationally intensive. In this paper, we extend the perturbed subsampling approach to the Cox proportional hazards model, a widely used method in survival analysis to address the statistical analysis of large-scale survival data. Specifically, we propose a perturbed subsampling algorithm for this model. The effectiveness of the proposed method is evaluated through simulation studies and real-data analysis.

## 1. Introduction

With the advancement of information technology and the widespread adoption of the internet, both the velocity and volume of data generation have increased dramatically, making large-scale data increasingly common.

Subsampling is a key method for large-scale data analysis. It works by drawing a smaller subset from the full data and performing estimation on this subset, thereby lowering computational cost. At its core, the method computes an optimal subsampling probability. A subset selected under this probability is designed to preserve the most information from the full population, resulting in parameter estimates that minimize the empirical mean squared error.

In recent years, research on subsampling methods has included: subsampling methods for linear models and generalized linear models [1,2,3] and subsampling methods for logistic regression models [4,5]. These studies are primarily designed for uncensored data, with their statistical inference frameworks typically relying on complete observational information. However, in survival analysis, due to the unique characteristics of the study subjects (such as patient loss to follow-up or events not occurring by the study cutoff), large-scale datasets containing substantial amounts of censored observations are highly common. Extensive research has been conducted on statistical analysis methods for such large-scale data with censored information. Numerous subsampling algorithms have been developed under different survival modeling frameworks. For example, in the context of functional quantile regression, subsampling strategies have been proposed by [6]. For the Cox proportional hazards model, subsampling methodologies have been examined by [7,8]. For applications involving rare events in Cox models, Ref. [9] introduced subsampling approaches based on both A-criteria and L-criteria. Furthermore, the subsampling approach has been extended to the additive hazards model by [10] and to the AFT model by [11].

Although the aforementioned subsampling methods can alleviate the computational burden posed by large samples, they require the prior derivation and calculation of optimal subsampling probabilities, a process that involves relatively complex theoretical analysis. Particularly when the model and data structure are more intricate, the derivation and computation can be time-consuming. To address this, Ref. [12] proposed a perturbed subsampling method, which introduces random weights and provides a way to estimate the asymptotic variance of subsample-based estimates using the full dataset.

Unlike simple random sampling, the perturbation subsampling method is distinguished by its capacity for statistical inference. Its key advantage lies in the introduction of a perturbation mechanism, which circumvents the need for pre-calculating optimal subsampling probabilities and, more importantly, provides a tractable approach for estimating the asymptotic variance. This feature is particularly advantageous for complex models, for instance, when the estimating function is non-differentiable, where traditional methods often struggle to compute asymptotic variances. In this paper, we validate the feasibility and effectiveness of this method within the important and intricate context of censored data. We propose a perturbed subsampling algorithm (Algorithm 1) and a repeatedly random perturbation subsampling algorithm (Algorithm 2). This work not only demonstrates the method’s superiority in specific models but also establishes a theoretical foundation for its broader application and further development.

## 2. Model and Notation

Let $X_{i}$ denote the *p*-dimensional covariate vector of the *i*th subject. $Y_{i}=\min\left\{T_{i},C_{i}\right\}$ represents the observed time, $T_{i}$ is the survival time and $C_{i}$ is the censoring time. Assume that $T_{i}$ and $C_{i}$ are independent given $X_{i}$. Let the total sample data be $D_{n}=\left\{\left(X_{i},T_{i},\Delta_{i}\right):i=1,2,\cdots ,n\right\}$, where *n* is the total sample size and $\Delta_{i}=I\left(T_{i}\leq C_{i}\right)$ is the censored indicator. The Cox proportional hazards model takes the form

(1) $$\begin{array}{c} \lambda \left(t|X_{i}\right)=\lambda_{0}\left(t\right)\exp\left(\beta^{T}X_{i}\right), \end{array}$$

where $\lambda (t|X_{i})$ is the conditional hazard function of $T_{i}$ given $X_{i}$, i=1,⋯,n. $\lambda_{0}\left(t\right)$ is an unspecified baseline hazard function.

Let $N_{i}\left(t\right)=I\left(\Delta_{i}=1,Y_{i}\leq t\right)$; then, the negative log-partial likelihood function for the Cox model is

(2) $$\begin{array}{cc} \ell (\beta ) & =-\frac{1}{n}\sum_{i=1}^{n}\int_{0}^{1}\left[\beta^{T}X_{i}-\log\left(\sum_{j=1}^{n}I\left(T_{j}\geq t\right)\exp\left(\beta^{T}X_{j}\right)\right)\right]dN_{i}\left(t\right)\\ \; & =-\frac{1}{n}\sum_{i=1}^{n}\Delta_{i}\left[\beta^{T}X_{i}-\log\left(\sum_{j=1}^{n}I\left(T_{j}\geq T_{i}\right)\exp\left(\beta^{T}X_{j}\right)\right)\right]. \end{array}$$

The maximum likelihood estimator of β is

$$\begin{array}{c} \hat{\beta }=\arg\min\left\{\ell (\beta )\right\}. \end{array}$$

For data with a large sample size *n*, the above estimation procedure is time-consuming. To avoid the derivation and computation of optimal subsampling probability, thereby saving computational time and improving estimation efficiency, we consider the following perturbation subsampling algorithm. First, to obtain a subsample of approximate size *r*, we independently generate *n* Bernoulli random variables with parameter $\pi_{n}=r/n$, where a value of 1 indicates that the corresponding sample point is selected into the subsample. Subsequently, to reconstruct the full-data objective function based on the subsample, we independently generate another set of nonnegative stochastic perturbation weights. These weights follow a completely known distribution with mean $1/\pi_{n}$. Through these two steps, a weighted subsample suitable for subsequent analysis is obtained, and the specific procedure is summarized in the following Algorithms 1 and 2.

**Algorithm 1** The perturbation subsampling algorithm**step1.** Draw a subsample. Let r≪n be the total subsample size of the subsample expected to be drawn from the total sample. Let $\pi_{n}=\frac{r}{n}$, and generate *n* independent and identically distributed random variables $\delta_{i}∼$Bernoulli$\left(\pi_{n}\right)$. Create a subsample consisting of records for which $\delta_{i}=1$. It should be noted that the number of subsamples actually drawn may not equal *r*. In fact, if we define $r^{*}$ as the size of the actually drawn subsample, then$$\begin{array}{c} r^{*}=\sum_{i=1}^{n}\delta_{i},\;E\left(r^{*}|D_{n}\right)=\sum_{i=1}^{n}E\left(\delta_{i}|D_{n}\right)=\sum_{i=1}^{n}\pi_{n}=r. \end{array}$$**step2.** Generate a random disturbance term. Generate *n* i.i.d. nonnegative random variables $\eta_{i}$ from a distribution with mean $\frac{1}{\pi_{n}}$ and variance $b_{n}^{2}$; these are referred to as the random disturbance terms.**step3.** Calculate the perturbed subsampling estimate. Calculate the random weights $\omega_{i}=\eta_{i}\delta_{i}$ based on the random disturbance terms. The perturbation-based subsampling estimator is$$\begin{array}{c} \tilde{\beta }=\arg\min_{\beta }\left\{-\frac{1}{n}\sum_{i=1}^{n}\omega_{i}\Delta_{i}\left[\beta^{T}X_{i}-\log\left(\sum_{j=1}^{n}I\left(T_{j}\geq T_{i}\right)\exp\left(\beta^{T}X_{j}\right)\right)\right]\right\}. \end{array}$$


In order to increase the convergence speed of the estimator, we propose to repeat the perturbation process described above *M* times. This leads to the repeated perturbation subsampling algorithm, described in the following steps:

**Algorithm 2** A repeatedly random perturbation subsampling algorithmFor m=1,⋯,M,**step1.** Generate *n* independent and identically distributed random variables $\delta_{mi}∼$Bernoulli$\left(\pi_{n}\right)$. Create a subsample consisting of records for which $\delta_{mi}=1$.**step2.** Generate *n* i.i.d. nonnegative random variables $\eta_{mi}$ from a distribution with mean $\frac{1}{\pi_{n}}$ and variance $b_{n}^{2}$; these are referred to as the random disturbance terms.**step3.** Calculate the perturbed subsampling estimate. Calculate the random weights $\omega_{mi}=\eta_{mi}\delta_{mi}$ based on the random disturbance terms. The perturbation-based subsampling estimator is$$\begin{array}{c} {\tilde{\beta }}_{m}=\arg\min_{\beta }\left\{-\frac{1}{n}\sum_{i=1}^{n}\omega_{i}\Delta_{i}\left[\beta^{T}X_{i}-\log\left(\sum_{j=1}^{n}I\left(T_{j}\geq T_{i}\right)\exp\left(\beta^{T}X_{j}\right)\right)\right]\right\}. \end{array}$$**step4.** Aggregate the estimation results: $$\begin{array}{c} {\tilde{\beta }}_{M}=\frac{1}{M}\sum_{m=1}^{M}{\tilde{\beta }}_{m}. \end{array}$$

## 3. Simulations

This section examines the performance of the aforementioned perturbed subsampling method with large-scale censored data. The true value of β is $\beta_{0}={\left(-1,-0.5,0,0.5,1\right)}^{T}$. We investigate three distinct scenarios with respect to the covariate ***X*** as follows:

Case I: A multivariate normal distribution (Normal), whose mean is zero and covariance is Σ. The covariance between *i* and *j* is $\Sigma_{ij}=0.5^{\left|i-j\right|}$, i,j∈{1,⋯,4}.

Case II: A multivariate *t* distribution with 10 degrees of freedom (T10), having the same mean and variance as in Case I.

The distributions in Case I and Case II are both symmetric, but the distribution in Case II is heavy-tailed. The censoring times follow a uniform distribution over (0,U), where *U* is chosen so that the censoring rate is about 20% or 60%. We perform 1000 replications of our simulations, with each replication having a sample size of $n=10^{6}$. Set the subsample size r∈{400,600,800,1000}. In addition, we consider the following two distributions for the perturbation term: Geometric($\pi_{n}$), Gamma($1/\pi_{n}$,1). Under various subsampling probabilities, the efficiency is assessed by evaluating the mean squared error (MSE) of ${\tilde{\beta }}_{M}$ over 1000 replications, where

$$\begin{array}{c} \mathrm{MSE}=\frac{1}{1000}\sum_{i=1}^{1000}{∥{\tilde{\beta }}_{M}^{\left(i\right)}-\beta_{0}∥}^{2}, \end{array}$$

**Figure 1:**
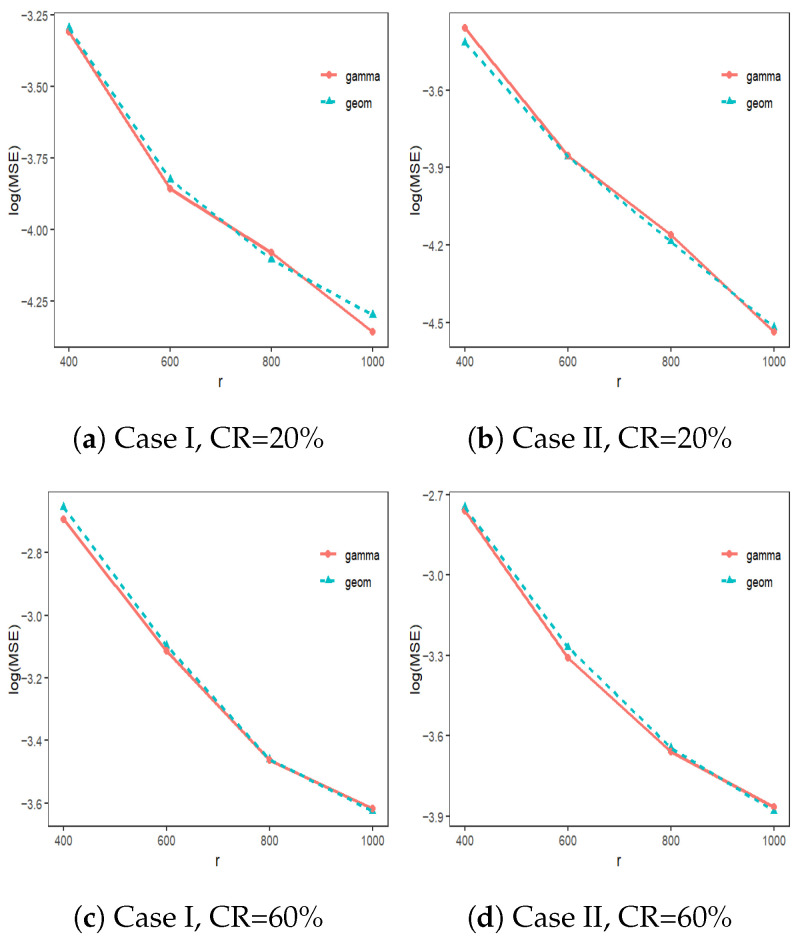
The MSEs for different subsampling methods.

Figure 1 shows the logarithm of MSEs for different distributions of perturbation terms in Case I and Case II. The results for the “Geometric” and “Gamma” perturbation terms are similar. Therefore, we will use the “Gamma” term in the subsequent analysis.

Additionally, we calculate the estimated bias (Bias), the empirical 95% coverage probability (CP) towards $\theta_{0}$, the estimated standard error (ESE) and the sampling standard error (SSE) to demonstrate the performance of our estimate with a finite sample size. The results for Case I and Case II are presented in Table 1 and Table 2. It can be seen from the results that as *r* increases, the values of Bias decrease. This indicates that our estimate is asymptotically unbiased. It can be seen that as *r* increases, the values of SSE and ESE both decrease, and are similar to each other. The coverage probabilities approximate the normal level.

In addition, we compare the computational efficiency of the perturbation subsampling method (Pert) proposed in this paper with the optimal subsampling methods described in references [7] (Sub_7_) and [8] (Sub_8_). Table 3 presents the computation time of different methods based on 1000 replications, under the condition of a 60% censoring ratio, with $r_{0}$ = 100, *r* = 500, and a sample size of $n=10^{6}$. $r_{0}$ is the initial subsample size in references [7,8]. Unlike the optimal subsampling methods in references [7,8], which require calculating optimal sampling probabilities, the proposed perturbation subsampling method eliminates this step, resulting in a slightly shorter computation time per iteration.

## 4. Real Data Analysis

In this section, we apply the proposed method on a gastric cancer dataset from the SEER (Surveillance, Epidemiology, and End Results) database (https://seer.cancer.gov/, accessed on 4 February 2026). We select patients diagnosed between 2005 and 2022, resulting in a total of 31,136 samples included for analysis, with a censoring rate of 67.72%. Covariates include age at diagnosis, race (1 = non-white), gender (1 = male), and year of diagnosis. The data were distributed across five nodes based on residence for processing, and different subsample sizes of r∈{400,600,800,1000} were investigated.

**Figure 2:**
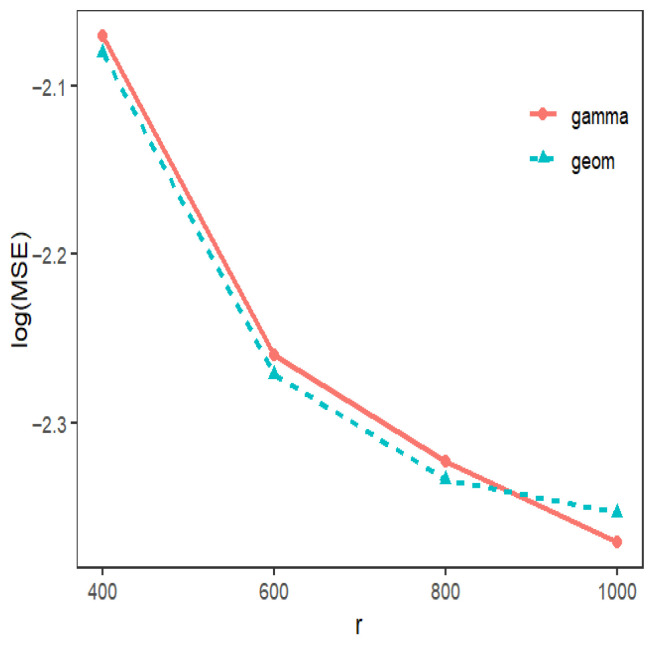
The MSEs for gastric data with different perturbation terms.

Figure 2 shows the logarithm of MSEs over 1000 replicates for different subsample methods. The results presented in Figure 2 are similar to those in Figure 1: as *r* increases, the MSEs decrease. Additionally, to illustrate the practical application of our method, we compute the full data estimation (MLE), as well as the subsample-based estimation using different perturbation terms. The results for over 1000 replicates with r=1000 are shown in Table 4. It is apparent that our method produces reliable estimates.

To further validate the performance of the proposed method under larger sample sizes, we applied it to the breast cancer dataset from the SEER database. We selected patients whose diagnosis year was between 1995 and 2020, comprising a total of 435,112 individuals, with a censoring ratio of 65.16%. We considered the following covariates: Age, Marital Status, Race, and Year of Diagnosis. Table 5 reports the parameter estimation results under different subsample sizes *r*. As *r* increases, the fluctuations in the estimates diminish and the values gradually stabilize, indicating that the proposed method exhibits desirable large-sample properties.

## 5. Discussion

This paper systematically investigates the perturbation subsampling method for the Cox proportional hazards model, aiming to address the issue of excessive computational burden associated with traditional optimal subsampling methods in the context of large-scale censored data. By introducing random perturbation weights, the proposed method avoids the pre-calculation of optimal subsampling probabilities, significantly reducing computational complexity while maintaining the statistical efficiency of parameter estimation. Theoretical analysis demonstrates that this perturbation mechanism not only effectively handles cases where the estimating function is non-differentiable but also provides a feasible approach for estimating asymptotic variances. Numerical simulation results further validate the superiority of the proposed method in terms of estimation accuracy and computational stability. In particular, when compared with classical optimal subsampling methods, the perturbation subsampling approach exhibits comparable or even superior performance.

Although this paper validates the effectiveness of the perturbation subsampling method within the framework of the Cox model, numerous directions warrant further exploration:

1. Relaxation of the Proportional Hazards Assumption: The proposed method relies on the proportional hazards assumption of the Cox model. Future research could investigate the adaptability and improvement strategies of the perturbation subsampling method when this assumption is violated (e.g., in the presence of time-varying effects or interactions), thereby extending its applicability in practical settings.
2. Handling Complex Data Structures: With advances in data collection technology, survival data are becoming increasingly complex. Future work could extend the perturbation subsampling method to scenarios involving distributed storage of large-scale survival data, addressing computational and inferential challenges when data cannot be processed centrally. Furthermore, mixture models with a cured fraction, informative censoring, and survival models incorporating time-varying covariates represent valuable directions for further investigation.
3. Extension to Other Censoring Types: This paper primarily focuses on right-censored data. Future research could generalize the proposed method to interval-censored data or left-truncated data scenarios. These data types are prevalent in clinical trials and epidemiological studies and pose new challenges for subsampling methodology.
4. Deepening Theoretical Properties: Further research into the asymptotic properties of perturbation subsampling under more general models, including theoretical guarantees in the context of non-smooth estimating functions and high-dimensional covariates, would provide a more solid theoretical foundation for its widespread application.

In summary, the perturbation subsampling method offers an efficient and robust tool for the statistical analysis of large-scale censored data. Its extension and application in more complex scenarios merit continued attention and in-depth investigation.

## Figures and Tables

**Table 1:** The proposed subsample estimate of β for Case I.

		CR = 20%				CR = 60%			
Para	r	Bias	ESE	SSE	CP	Bias	ESE	SSE	CP
$\beta_{1}$	400	0.0736	0.0796	0.0857	0.915	0.1064	0.1157	0.1247	0.890
	600	0.0557	0.0659	0.0659	0.910	0.0810	0.0896	0.1009	0.915
	800	0.0461	0.0559	0.0556	0.935	0.0625	0.0769	0.0750	0.945
	1000	0.0397	0.0496	0.0486	0.940	0.0565	0.0684	0.0676	0.940
$\beta_{2}$	400	0.0655	0.0795	0.0797	0.930	0.0877	0.1140	0.1106	0.940
	600	0.0463	0.0633	0.0588	0.940	0.0777	0.0895	0.0962	0.910
	800	0.0474	0.0536	0.0581	0.910	0.0640	0.0781	0.0785	0.945
	1000	0.0406	0.0491	0.0500	0.950	0.0584	0.0665	0.0720	0.920
$\beta_{3}$	400	0.0643	0.0755	0.0813	0.925	0.0884	0.1084	0.1076	0.955
	600	0.0513	0.0594	0.0623	0.935	0.0645	0.0863	0.0829	0.940
	800	0.0401	0.0520	0.0505	0.935	0.0619	0.0741	0.0763	0.925
	1000	0.0373	0.0457	0.0471	0.940	0.0601	0.0653	0.0741	0.895
$\beta_{4}$	400	0.0642	0.0800	0.0823	0.930	0.0922	0.1137	0.1132	0.925
	600	0.0472	0.0627	0.0605	0.935	0.0694	0.0884	0.0902	0.940
	800	0.0459	0.0540	0.0573	0.910	0.0609	0.0787	0.0782	0.925
	1000	0.0414	0.0480	0.0534	0.905	0.0620	0.0662	0.0751	0.920
$\beta_{5}$	400	0.0740	0.0814	0.0835	0.915	0.0860	0.1138	0.1058	0.950
	600	0.0569	0.0648	0.0672	0.935	0.0798	0.0910	0.0926	0.930
	800	0.0482	0.0561	0.0590	0.905	0.0670	0.0781	0.0806	0.915
	1000	0.0427	0.0496	0.0518	0.920	0.0600	0.0675	0.0723	0.925

**Table 2:** The proposed subsample estimate of β for Case II.

		CR = 20%				CR = 60%			
Para	r	Bias	ESE	SSE	CP	Bias	ESE	SSE	CP
$\beta_{1}$	400	0.0666	0.0771	0.0778	0.905	0.0895	0.1056	0.1012	0.920
	600	0.0495	0.0605	0.0608	0.935	0.0673	0.0838	0.0828	0.920
	800	0.0468	0.0524	0.0574	0.915	0.0609	0.0719	0.0736	0.920
	1000	0.0377	0.0467	0.0468	0.915	0.0556	0.0625	0.0670	0.925
$\beta_{2}$	400	0.0642	0.0733	0.0775	0.915	0.0829	0.1044	0.1071	0.915
	600	0.0541	0.0571	0.0661	0.880	0.0599	0.0798	0.0747	0.950
	800	0.0486	0.0493	0.0596	0.865	0.0585	0.0676	0.0688	0.930
	1000	0.0355	0.0442	0.0438	0.925	0.0472	0.0618	0.0612	0.945
$\beta_{3}$	400	0.0650	0.0676	0.0769	0.920	0.0856	0.0980	0.1081	0.890
	600	0.0459	0.0548	0.0579	0.935	0.0671	0.0801	0.0846	0.945
	800	0.0392	0.0473	0.0491	0.930	0.0544	0.0661	0.0678	0.940
	1000	0.0307	0.0428	0.0380	0.955	0.0472	0.0587	0.0574	0.945
$\beta_{4}$	400	0.0635	0.0749	0.0802	0.895	0.0917	0.1038	0.1127	0.935
	600	0.0523	0.0585	0.0671	0.910	0.0699	0.0800	0.0876	0.910
	800	0.0423	0.0508	0.0516	0.930	0.0535	0.0689	0.0660	0.950
	1000	0.0359	0.0442	0.0446	0.940	0.0500	0.0631	0.0635	0.930
$\beta_{5}$	400	0.0724	0.0784	0.0870	0.880	0.0948	0.1068	0.1026	0.945
	600	0.0540	0.0607	0.0655	0.925	0.0733	0.0829	0.0855	0.925
	800	0.0458	0.0536	0.0563	0.925	0.0619	0.0710	0.0741	0.925
	1000	0.0411	0.0472	0.0501	0.925	0.0550	0.0629	0.0672	0.925

**Table 3:** Average CPU time (unit: seconds).

Method	Pert	Sub_7_	Sub_8_
Time	1.061	1.410	1.270

**Table 4:** The full data estimation (MLE) and the subsample-based estimation for *r* = 1000.

Risk	MLE	Gamma	Geom
Age	0.0512	0.0566	0.0560
Race	−0.0493	−0.0520	−0.0545
Sex	0.0240	0.0207	0.0227
Year	−0.0919	−0.0933	−0.0931

**Table 5:** The subsample-based estimation for r∈{400,600,800,1000}.

	r=400		r=600		r=800		r=1000	
Risk	Gamma	Geom	Gamma	Geom	Gamma	Geom	Gamma	Geom
Age	0.8594	0.8578	0.8632	0.8650	0.8635	0.8625	0.8542	0.8553
Marital	−0.0821	−0.0818	−0.0865	−0.0864	−0.0846	−0.0835	−0.0822	−0.0830
Race	0.0963	0.0837	0.0696	0.0750	0.0969	0.0970	0.0974	0.0966
Year	−0.0565	−0.0583	−0.0517	−0.0521	−0.0598	−0.0606	−0.0539	−0.0542

## Data Availability

The gastric and breast cancer data were obtained from the SEER program website (https://seer.cancer.gov).
